# A framework for adequate nourishment: balancing nutrient density and food processing levels within the context of culturally and regionally appropriate diets

**DOI:** 10.1093/af/vfae032

**Published:** 2025-04-05

**Authors:** Frédéric Leroy, Ty Beal, Nel de Mûelenaere, Stefaan De Smet, Frits Heinrich, Lora Iannotti, Bradley Johnston, Neil Mann, Andrew Mente, Alice Stanton

**Affiliations:** Industrial Microbiology and Food Biotechnology (IMDO), Faculty of Sciences and Bioengineering Sciences, Vrije Universiteit Brussel, Brussels, Belgium; Global Alliance for Improved Nutrition (GAIN), Washington, DC, USA; Institute for Social, Behavioral and Economic Research, University of California, Santa Barbara, CA, USA; Interdisciplinary Historical Food Studies (FOST), Faculty of Languages and Humanities, Vrije Universiteit Brussel, Brussels, Belgium; Laboratory for Animal Nutrition and Animal Product Quality, Faculty of Bioscience Engineering, Ghent University, Ghent, Belgium; Industrial Microbiology and Food Biotechnology (IMDO), Faculty of Sciences and Bioengineering Sciences, Vrije Universiteit Brussel, Brussels, Belgium; Interdisciplinary Historical Food Studies (FOST), Faculty of Languages and Humanities, Vrije Universiteit Brussel, Brussels, Belgium; E3 Nutrition Lab, Brown School, Washington University in St. Louis, St. Louis, MO, USA; Department of Nutrition, College of Agriculture and Life Sciences, Texas A&M University, College Station, TX, USA; Department of Epidemiology and Biostatistics, School of Public Health, Texas A&M University, College Station, TX, USA; Department of Health Research Methods, Evidence and Impact, McMaster University, Hamilton, Ontario, Canada; School of Exercise and Nutrition Science, Deakin University, Geelong, Australia; Population Health Research Institute and Hamilton Health Sciences, Hamilton, Ontario, Canada; Department of Health Research Methods, Evidence and Impact, McMaster University, Hamilton, Ontario, Canada; School of Pharmacy and Biomolecular Sciences, Royal College of Surgeons in Ireland, University of Medicine and Health Sciences, RCSI Education & Research Centre, Beaumont Hospital, Dublin, Ireland

**Keywords:** animal-source foods, dietary guidelines, nutrient security, ultra-processed foods

**Implications**

- There are many dietary patterns and habits for the human omnivore to achieve adequate nourishment, often beyond what conventional nutritional recommendations and models suggest. Yet, there are also limits to this dietary flexibility, contingent on nutrient density and food processing levels.
- The nutrient density of a diet is usually improved by incorporating animal-source foods beyond a threshold, suggested to be at one-fourth to one-third of the caloric intake. Below this threshold, nutrient deficiency risks become a concern and require careful consideration. In contrast, consuming a high share of animal-source foods comes with contentious debates related to the risk of chronic diseases.
- Minimally processed foods are recommended as the preferred dietary option, but this should not compromise nourishment potential. Especially when plant-based foods dominate dietary intake, processing steps are needed to remove phytotoxins and improve nutrient bioavailability. However, excessive processing levels result in loss of food quality, whereas dietary patterns dominated by ultra-processed foods are likely harmful.
- This article focuses on the health aspects of human diets, without addressing concerns related to environmental impact or animal welfare. Such concerns can nonetheless be met, to some degree, if dietary guidance sufficiently embraces flexibility. This can be done by encouraging culturally appropriate diets while allowing for the self-selection of a variety of nutrient-dense, satiating foods of a mainly minimally processed nature, based on personal and cultural needs, values, and preferences. The concept of adequate nourishment is particularly important for populations with unique nutritional requirements, such as young children, pregnant and lactating women, and older adults.

## Introduction

This article constitutes an interdisciplinary effort combining expertise on such topics as nutritional epidemiology, nutrient security, clinical nutrition, evidence-based medicine, cultural food studies, food history, archaeobotany, and food technology. It aims to synthesize a part of the timeliest conversations on human diets and adequate nourishment, in particular, the roles of nutrient density (and its relation to the dietary animal–plant ratio) and food processing levels (including the much-debated issue of ultra-processed foods). By balancing these dimensions, optimal dietary domains will be proposed with a freedom to operate in manners that are respectful of food sovereignty, while upholding the potential for diverse dietary recommendations. Such flexibility should allow individuals to align their nutritional status with their idiosyncratic perspectives and values based on regional, culinary, and cultural appropriateness, ethics, and sustainability, and within practical and budgetary constraints.

We wish to stress that the focus of the article is on nourishment and that we do not venture into other aspects of ultra-processed foods and animal production that are also critical to address, including impact on food politics, biodiversity, climate, and animal welfare, but that are beyond the scope of the current paper.

## From “Healthy Diets” to Adequate Nourishment

Ongoing nutritional debates in Western high-income countries frequently revolve around two food categories, namely processed foods, in particular, the “ultra-processed” variants (Astrup and Monteiro, 2022), and animal-source foods, especially red meat, processed meat, and animal fat (Barnard and Leroy, 2020). These debates are not new; they were already part of the conversation led by diet reform movements prior to and during the Progressive Era in the United States (1890s to 1920s). The latter period was characterized by an interest in *scientific management*, fueled by both progressive ideals and wartime food rationing (in 1917), with the aim to increase efficiency and output and to improve public hygiene and health (Biltekoff, 2013; Veit, 2013). This implied the enforcement of standards and rules based on new scientific insights about calories and vitamins, since it was assumed that citizens were unable to govern their own bodies, leading to suboptimal societal performance (Biltekoff, 2013). Reformists advocated for governmental interference in American diets to steer public health toward an optimum, preferring a bland but “rational” New England diet over food choices driven by tradition or taste. Part of the movement, whether out of secular or religious motives, disapproved of red meat and some commonly purchased processed ingredients and foods (sugar and white bread), which were seen as harmful luxuries, while eulogizing whole grains, nuts, and fruits (Shprintzen, 2003). The First World War introduced additional reasons to restrict red meat, butter, sugar, and refined wheat at home, but this time from an economic, geopolitical, and logistical perspective (shipping food aid to Europe in a stable and efficient manner), which resulted in governmental calls for responsible citizenship, by stimulating meatless days and the consumption of wholegrains, while also praising “modern” processed foods like margarine, peanut butter, etc. (Veit, 2013). Reformist views were propagated by the discipline of domestic science, or home economics, institutionalized in the first dietetic associations, and taken up by the American progressive middle class (Leroy and Hite, 2020). In practice, this implied that dietary advice could now be used to delineate social norms and impose middle-class values through what appeared to be the neutral language of science (Biltekoff, 2013).

After decades of shifting priorities and lobbying by opposing interests to either restrain or promote the role of specific food groups, dietary guidelines have attempted to limit the full human omnivore spectrum to what is deemed a healthy eating pattern. Such advice cannot be separated from its historical *conditions of possibility*, i.e., the ways in which it emerged as knowledge at the intersection of institutions, industry, discourse, and social practices, thereby gaining acceptance within society. The constructed notion of “eating healthy,” therefore, overlaps with that of “eating right” (Biltekoff, 2013; Veit, 2013).

This prelude is not merely of historical interest; it illustrates how dietary opinions and beliefs are formed and reshaped over time, often under the influence of factors and processes outside of nutritional science itself. This may also help to explain the origins and nature of the *healthy user bias* in populations that are commonly the focus of observational studies in the nutritional epidemiology of chronic disease (Leroy and Hite, 2020). Healthy user bias occurs when individuals who engage in a particular health behavior also engage in other healthy behaviors that independently contribute to better health outcomes, distorting the apparent effects of the health behavior studied. The cultural contingency of such a bias may form the basis of the deviations in health outcomes associated with, for instance, red meat intake in North America, in contrast to other regions, or when assessed globally (Johnston et al., 2023; Mente et al., 2023). Healthy user bias is but one of the many challenges faced by nutritional epidemiology in its quest to become “more rigorous and informative” (Brown et al., 2023). Moreover, the discourse has become saturated with references to the now semantically eroded notion of “healthy diets” and a renewed emphasis on *adequate nourishment* may be needed (Provenza, 2018). The latter is not limited to reducing cardiometabolic risk or ensuring nutrient intake; it also implies wholesomeness, satiety, vitality, and alignment with personal, biological, and cultural needs.

## Contemporary Concerns

Despite decades-long efforts toward establishing “healthy” dietary guidelines and food policies worldwide, there has been a steady increase in the prevalence of diet-related noncommunicable diseases. In the United States, only 7% of adults are still displaying optimal cardiometabolic health (O’Hearn et al., 2022). Given the increase in chronic diseases, it is imperative to explore whether a new dietary path forward can be charted that is both adaptable and respectful of cultural, regional, and individual choices, while also capturing the key contributors to proper nourishment. When it comes to the role, efficacy, acceptability, and understanding of dietary guidelines, three interrelated points of attention can be identified.

1. Some dietary models have been criticized for being paternalistic or neocolonial, due to their insistence on the universal validity of Western-centric approaches (Burnett et al., 2020). In response, dietary guidelines are increasingly formulated with the aim of better aligning with culturally appropriate solutions (Herforth et al., 2019). Within Southern Europe, for instance, valid alternatives to a now globalized standard Mediterranean-style diet can be found, such as the Atlantic diet typified by regular consumption of red meat and processed pork (Carballo-Casla et al., 2024). There are, indeed, multiple ways to adopt a nourishing diet (Kuhnlein et al., 2009); many of these approaches (e.g., low-carb diets, certain indigenous diets, or the very diets on which *Homo sapiens* evolved) largely conflict with standard dietary guidelines.
2. Each body is unique, with varying nutrient requirements and metabolic responses (Berry et al., 2020). This is further complicated by the multitude of interactions between the plethora of nutrients that make up meals and the fact that nutritional requirements also depend on the specific type of diet followed by an individual (for instance, when diets lead to more oxidative stress, higher amounts of antioxidants may be needed). The notion of a generalized optimal diet is thus illusory and may undermine the efficacy of the body’s “nutritional wisdom,” including an individual’s capacity to self-select foods and balance nutrient intake levels based on innate and acquired physiological feedback. The validity of the latter mechanism has been extensively documented in animals (Provenza, 2018), but there is no clear reason as to why humans would differ from other animals in this respect (Davis, 1939), except for cultural maladaptation, wherein the consumption of modern (ultra-processed) foods may override natural feedback signals of satiety and hunger (Hall et al., 2019).
3. The validity of what tends to be accepted dietary advice is limited by the fact that the underpinning evidence is largely based on observational studies (Brown et al., 2023). The certainty of such evidence should mostly be qualified as low to moderate at best, especially when using GRADE certainty of evidence assessments, as shown for the health risk effects of red and processed meats (Johnston et al., 2019, 2023). Marantz et al. (2008) have argued that low-certainty advice may have led to an overly zealous avoidance of dietary fat, while concluding that “when adequate evidence is not available, the best option may be to issue no guideline.” Consensus-based guidelines, as opposed to evidence-based guidelines, are common in the nutritional domain and tend to allow panels to be less rigorous, generating inappropriately strong recommendations (Yao et al., 2021). When there is low-quality, uncertain evidence for the potential benefits and harms linked to diet, guideline recommendations should be weak, conditional, and more pluralistic. In addition, guidelines should be based on the health-related values and preferences of the guideline users (e.g., members of the public), a component of evidence-based practice that is regularly neglected (Yao et al., 2021).

**Figure 1.**
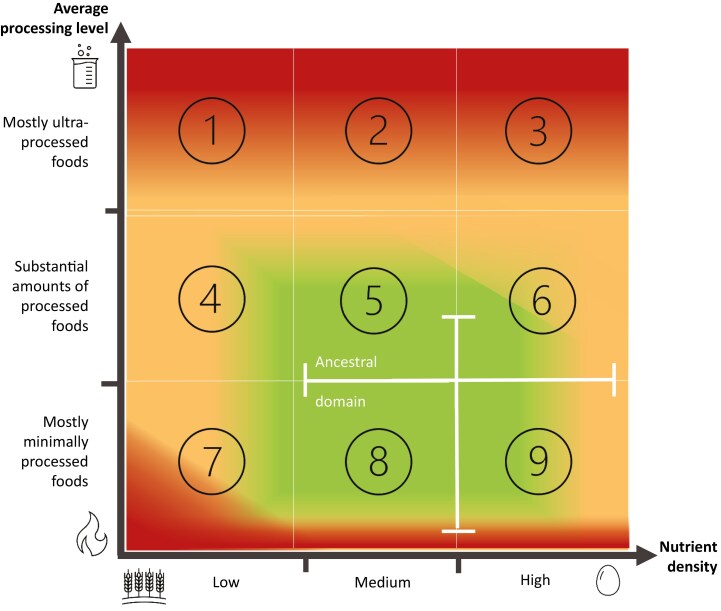
Conceptual summary, based on the authors’ interpretation of the literature, of the effects of processing level and nutrient density on the nourishment potential and overall health impact of diets (red: likely harmful; orange: possibly risky; green: likely benign) into nine different combinations (1 to 9). The white lines indicate the likely ancestral dietary domain, corresponding with the human evolutionary dietary background.

**Figure 2.**
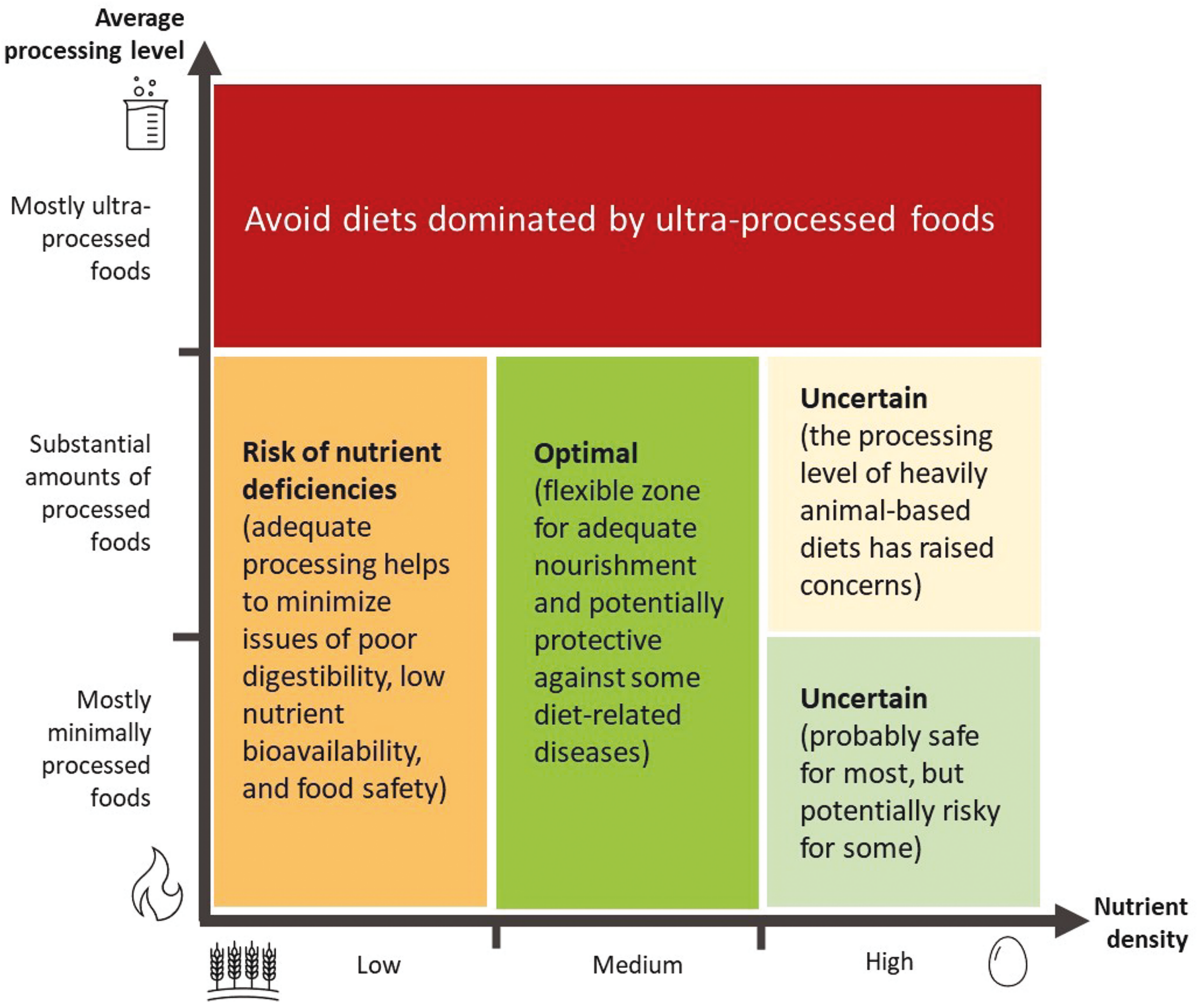
Simplified conceptual summary, based on the authors’ interpretation of the literature, of the effects of processing level and nutrient density on the nourishment potential and overall health impact of diets.

Given this combination of uncertain evidence and variable needs, the objective of this article’s dietary framework is to explore options for adequate nourishment while maintaining flexibility. To do so, a dietary space is proposed based on 1) nutrient density, correlating with the trophic level or animal–plant ratio to at least some degree, 2) the diet’s average food processing level, and 3) the potential to nourish adequately (Figures 1 and 2). Note that such estimates are based on the authors’ interpretation of the literature, and thus subject to future refinements.

## Allowing for Dietary Flexibility

### The human omnivore

Humans are exceptionally flexible omnivores, which allowed them to colonize an astonishing variety of ecological niches, from the equator to the arctic, and including such diverse ecosystems as savannas, rainforests, mountains, tundra, and deserts. Such diversity is mirrored in the dietary practices of ancestral-type populations, for instance, with respect to the discrepancies in the intake of animal-source foods. However, when and where the ecological baseline allows it, animal-source foods are foundational to hunter–fisher–gatherer diets. In a study of modern foragers by Cordain et al. (2000), three-fourths of the communities derived at least half of their energy from animals. Only a small minority of the communities investigated (4%) obtained less than one-third of their calories from animal-source foods, and none consumed less than 14%. Taken together, modern foragers obtain about two-thirds of their calories from hunting and fishing on average (Cordain et al., 2000), and half in warm climates (Pontzer and Wood, 2021). For Paleolithic hunter–fisher–gatherers, the range of animal foods has similarly been estimated at 30% to 70% of the caloric intake (Kuipers et al., 2010). Evolutionary dietary patterns characterized by high levels of animal-source foods have also been shown for children specifically (Iannotti et al., 2022).

Ecologically, the effects of seasonality and climate may have favored more carnivorous diets in some contexts (Zhu et al., 2021). The high energy density of animal-source foods in comparison to the limited expenditure of effort required (per calorie) to obtain them, especially in comparison with most (small-seeded or leafy) plant-based food sources prior to energy-dense staple crops becoming available, is often held responsible for this preference, although more complex dynamics of reaching nutritional adequacy beyond caloric equilibrium may also be at play (Leroy et al., 2023). An emerging body of Paleolithic archaeobotanical evidence, including some of the earliest finds of processed food remains, composed of wild cereals, pulses, and nuts (Kabukcu et al., 2023), does warrant some caution against universally underestimating the role of plants in early diets. Moreover, while plants may have played a smaller role in terms of caloric intake, it should be noted that both in current and past ancestral-type populations, and to a lesser extent in traditional farming communities, the consumption of low-caloric but nutrient-dense wild plants is much more frequent and diverse in terms of taxa than is common in modern Western diets (Oduor et al., 2023).

### The role of technology

A key strategy that has allowed the human species to vastly expand its dietary breadth is the adoption of food processing technologies. Through practices such as the cutting, chopping, pounding, soaking, sprouting, fermenting, salting, drying, and heating of raw materials, our ancestors could incorporate various foods into their diets that would otherwise have been inaccessible, unpalatable, poorly digestible, unhygienic, or even toxic (Kuhnlein et al., 2009).

While the processing of animal-source foods primarily aims to enhance shelf-life and ensure microbial food safety, the fermentation and “putrefaction” of fish, meat, and fat by early humans and contemporary hunter-gatherers in the (sub)arctic to “predigest” raw food without using fuel is an important exception (Speth, 2017). The processing of plants, however, holds even much greater significance, as many plants inherently contain toxins, such as lectins, cyanogenic glycosides, and alkaloids, as well as antinutrients such as phytates and oxalates. This explains why many traditional plant food processing techniques had their likely origin prior to the emergence of agriculture (e.g., the roasting or soaking of acorns, drying of mushrooms, boiling of tubers and roots, and fermentation of various plant materials). Most of these techniques persist until this day and have been supplemented with modern high-tech options, which further enhance dietary flexibility, convenience, and food safety.

Critical modern options include the use of dietary fortification, biofortification, and supplementation, which offer the opportunity to enhance the nutrient content of specific foods (or crops, in the case of biofortification). This approach enables humans to explore diets with reduced reliance on animal-source foods compared to ancestral-type diets. Since animal-source foods provide key micronutrients and bioactive compounds that are less available or unavailable in plants (e.g., iron, zinc, vitamins B12 and D, long-chain omega-3 fats, and creatine), fortifying plant-derived foods can partially fulfill this nutritional role.

### Limits to flexibility?

Despite the natural adaptability of the human biological constitution and the advancement of processing technologies, there are inherent limitations to this flexibility. Two concerns arise:

1. Simply concocting a balanced mix of macronutrients fortified with essential nutrients may not suffice for proper nourishment (Provenza, 2018). Such a reductionist viewpoint overlooks the intricate nature of healthy dietary patterns, which involve matrix effects and a plethora of bioactive compounds that may not be deemed “essential” but nevertheless contribute to health (Leroy et al., 2023). Drastically limiting animal-source foods hinders the adoption of whole-food strategies, which are nutritionally and culturally preferable, given that various nutrients are difficult to obtain naturally in adequate quantities from plants. In contrast, very high consumption levels of animal-source foods may well be favorable for nutritional adequacy but raise concerns with respect to the risk of noncommunicable diseases. There is thus a need to identify what it means to have either “too little” (Leroy et al., 2023) or “too much” (Johnston et al., 2023) animal-source foods in one’s diet.
2. While food technology is valuable in increasing dietary flexibility, its use becomes counterproductive when geared toward the production of hyperpalatable foods that promote overeating, commonly referred to as ultra-processed foods. The latter are mostly formulated using cheap industrial ingredients, with multiple additives, and manufactured using high-intensity processes that disrupt the food matrix and may lead, at least in some cases, to the loss of valuable nutrients and the formation of harmful compounds (Scrinis and Monteiro, 2022). Once again, it needs to be explored how either “too little” or “too much” processing can restrict dietary flexibility (Astrup and Monteiro, 2022).

## Nutrient Density Level

### Context: reasons for variability

While there are concerns that the risk of chronic diseases may increase at high intake levels of animal-source foods, the most nutrient-dense foods and top sources of commonly lacking nutrients are largely of animal origin, including red meat, fish, seafood, eggs, and milk, even if certain plants such as dark green leafy vegetables also rank highly (Beal and Ortenzi, 2022). Animal-source foods, which are typically situated at the higher trophic level of the dietary spectrum, bring in high-quality protein, essential fatty acids (DHA and EPA), micronutrients (haem iron, zinc, calcium, iodine, retinol, vitamins D3 and B12, etc.), and bioactive compounds (choline, taurine, creatine, carnitine, carnosine, etc.), several of which are difficult to obtain from plants, or are absent in plants altogether (Leroy et al., 2023).

The animal–plant ratio has varied substantially throughout history. Lower animal–plant ratios are obvious when comparing contemporary to ancestral-type dietary models (Table 1), a situation which is further complicated by the reduced nutrient content of modern plant foods due to crop yield improvements (Heinrich and Erdkamp, 2018) and climate change (Myers et al., 2017). On average, this has resulted in a lower nutrient density of today’s diets when compared to evolutionary levels (Dewey, 2013). Historically, the adverse effects of the lowering of this ratio, in addition to several other factors, on nutrient security during the Neolithic dietary transition have been well documented (Armelagos et al., 1991). Agriculture did not initially lead to a better-fed population, but only to more people. The so-called *secondary product revolution*, which introduced (processed) dairy and eggs and made animal protein more accessible, later somewhat countered this, as can be deduced from skeletal remains. While human stature is dependent on complex factors, it is strongly linked to the intake of animal-source foods, given their role in the physical development at a young age and their excellent digestible indispensable amino acid scores (Leroy et al., 2023). Populations that were comparatively tall are typically interpreted as having enjoyed diets higher in animal protein and having had a better nutritional status (Koepke and Baten, 2005).

**Table 1.** Animal-source foods as percent of total calories

Percent	Ancestral-type data
14 to 99 (median 66 to 75)	Modern hunter-gatherers (Cordain et al., 2000)
50	Modern hunter-gatherers in warm climates (Pontzer and Wood, 2021)
30 to 70	Pleistocene diets (Kuipers et al., 2010)

Percent	Current data per region, based on food balance sheets (source: FAOSTAT, https://www.fao.org/faostat/en/#data/FBS)
35 to 41	Individual countries: Estonia (41%), Denmark (41%), Iceland (38%), Hong Kong (38%), Mongolia (38%), Finland (38%), Netherlands (37%), Belgium (36%), France (35%), Ireland (35%)
20 to 34	Western Europe (34%), Northern Europe (30%), Australia-New Zealand (30%), Northern America (28%), Eastern and Southern Europe (27%), Central Asia (26%), South America (25%), Central America (20%)
10 to 19	Eastern Asia (19%), World average (18%), Asian and Western Asian average (16%), Southern Africa (17%), Caribbean (14%), Southern Asia (13%), South-Eastern Asia (11%), Northern Africa (11%)
0 to 9	Eastern Africa (7%), Middle and Western Africa (5%)

Finally, while the common environmental argument that eating at lower trophic levels leads to a more efficient and sustainable food system makes a point, from a nutritional perspective, it overlooks the fact that the physiology of *Homo sapiens* is evolutionary adapted to nutrient-rich diets and depends on them. In general, higher animal–plant ratios facilitate meeting adequate essential nutrition targets, even if they may not guarantee them. There is, therefore, a limit to the human ability to eat at a lower trophic level, in combination with the fact that animals are capable of converting foods of low nutritional value and nonedible resources, such as agri-food industry byproducts or forages from nontillable pastures, into products that humans can metabolically access and benefit from.

### Low nutrient density

At low animal–plant ratios, the density of priority nutrients in a diet tends to decrease. A reanalysis of the EAT-Lancet diet, a plant-dominated diet sourcing only 14% of its calories from animals, showed a shortfall in iron, zinc, calcium, and vitamin B12 (Beal et al., 2023). In the absence of fortification and supplementation, a doubling of calories from animal origin to 27% would be needed to meet the nutritional recommendations of adult populations. This is not only similar to the threshold below which increased micronutrient risk is seen in current national diets worldwide (Nordhagen et al., 2020), but also corresponds with what can be concluded from an evolutionary perspective when looking into ancestral diets (“Allowing for dietary flexibility” section). Alternatively, there is evidence to suggest that about half of the total protein intake by adults must be animal-based to meet nonprotein nutrient-based recommendations (Vieux et al., 2022).

A threshold of about 25% to 30% of kcal from animal-source food (or half of the protein intake) is of course not a definite and unambiguous one, as the threshold would be contingent on the nutritional quality of both the animal- and plant-derived foods involved. For instance, the regular consumption of liver may allow for a lower share of animal-source foods to meet nutritional adequacy of priority nutrients, compared to a diet dominated by chicken filet. A similar argument can be made when comparing diets that incorporate nutrient-dense leafy vegetables with ones that are heavily based on starchy staples. Furthermore, Western populations that meet a 25% to 30% kcal threshold on average (Table 1) nonetheless suffer from both poor cardiometabolic health (O’Hearn et al., 2022) and micronutrient deficiencies, especially in populations with elevated needs (Devarshi et al., 2021). Such deficiencies may result in a variety of diseases, including anemia (iron and vitamin B12), osteoporosis (calcium and vitamin D), neuropathy (vitamin B12), hypothyroidism (iodine), and poor immunity (e.g., zinc deficiency syndrome). If ancestral-type populations present sustained cardiometabolic health and nutritional adequacy at the 25% to 30% level, or even somewhat below, this should be ascribed to the higher nutrient density of their diets compared to contemporary variants. This could be due to the greater proportion of traditionally processed rather than ultra-processed foods, or to the much greater qualitative diversity of the (wild) plant sources consumed at the same quantitative proportion (“Allowing for dietary flexibility” section). Nonetheless, there seems to be a reasonable case to argue that, for the general populace in the (post-)industrial world, nutrient adequacy is more easily achievable when one-fourth to one-third of calories are of animal origin. This can be ascribed to the fact that animal-source foods are typified by a higher density and bioavailability of protein and micronutrients that are commonly limiting in global diets (for a more detailed discussion on this topic, see Leroy et al., 2023). Below that threshold, it is recommended to carefully monitor dietary adequacy and supplement or fortify limiting nutrients.

### Medium nutrient density

“Medium nutrient density” should be understood as the nutritional domain encompassing most of the ancestral dietary variants, where relatively variable amounts of nutritious and satiating foods, consisting of proteinaceous animal-source foods and micronutrient-dense plants (e.g., dark leafy vegetables), are complemented with foods that have moderate (e.g., pulses and quinoa) or even low nutrient densities (e.g., tubers, fruits, and most cereals; Beal and Ortenzi, 2022). Such diets appear to be protective against noncommunicable diseases (Carballa-Casla et al., 2024), provided that foods are of a minimally or moderately processed nature (when ultra-processed foods are dominant, a separate analysis is needed; see below). A systematic review and network meta-analysis of 40 randomized trials, comparing 7 multimodal programs with varying dietary patterns, suggested that those with substantial amounts of animal-source foods (e.g., Mediterranean-style diet programs) have the best available evidence for reducing the risk of all-cause and cardiovascular mortality, stroke, and myocardial infarction based on moderate certainty evidence, while dietary programs with a more vegetarian pattern showed little to no benefit based on low- to very low-certainty evidence (Karam et al., 2023). The latter study does not lead to definitive conclusions on animal–plant ratios, but based on its robustness, does suggest that the evidence for protective effects with traditional, regional diets containing animal protein and fats is superior to that of vegetarian diets.

Comprehensive data from the global PURE cohort and 5 independent studies on a total of 245,000 people from 80 countries has shown that a diet comprised of high amounts of fruit, vegetables, nuts, legumes, fish, and whole-fat dairy is associated with lower incidence of cardiovascular disease and mortality (Mente et al., 2023). The findings from this uniquely large and diverse international study were consistent in individuals with or without vascular disease and in all world regions, especially in countries with lower income where intakes of nourishing foods containing priority micronutrients were lowest. Even in adults, low intake of such cornerstone foods (and potentially undernutrition) rather than excess intake of foods associated with noncommunicable diseases may be the major problem with diet in relation to mortality and cardiovascular disease globally. The PURE diet score was designed for population-level recommendations and, given the consistency of the results in different settings, can be used as the basis for recommendations globally, in view of a reduced mortality and cardiovascular disease risk, and then modified for each region based on the specific types of foods that are available and affordable in each region. This can at the same time address the continuing and severe problem of undernutrition in many countries or even poorer segments of high-income countries, as various of these protective foods are also among the most dense in micronutrients.

### High nutrient density

The upper-density nutrient level can only be met with high proportions of animal-source foods, particularly when processing levels are minimal. The concern here is that elevated intakes of animal-source foods, rich in saturated fats, particularly red meat, eggs, and dairy, have been associated with increased risk of noncommunicable diseases. However, more recently, the evidence linking animal-source foods to detrimental effects on human health has been questioned (Johnston et al., 2019, 2023; Astrup et al., 2020). Whereas eggs and poultry meat have neutral impacts on mortality and morbidity, the consumption of at least two helpings of whole-fat dairy a day is even associated with protection against obesity, cardiovascular events, and some cancers (Dehghan et al., 2018; Feng et al., 2022). Up to three servings of seafood a week are associated with both brain and cardiovascular protection (Mohan et al., 2021). With respect to unprocessed red meat, recent systematic reviews concluded that the possible absolute effects of its intake on cardiovascular and cancer outcomes are small and uncertain (Johnston et al., 2019, 2023; Lescinsky et al., 2022). Importantly, the relationship is not necessarily causal. When relatively large quantities of red meat (50 to 100 g daily on average) are consumed as part of a diet rich in whole or minimally processed foods, risks appear to diminish considerably or disappear (Maximova et al., 2020; Mente et al., 2023).

However, diets that consist of very large quantities of animal-source foods tend to be highly caloric and risk suboptimal levels of fiber, certain micronutrients, and phytochemicals more readily sourced from plants. Although such diets can be of interest for their therapeutic potential, more scientific data are required to robustly evaluate their risks and benefits (Protogerou et al., 2021). For instance, individuals prone to hemochromatosis may have to control red meat intake (or opt for compensatory interventions), whereas high saturated fat intake may negatively affect some risk markers in high responders (Barnard and Leroy, 2020).

## Aspects of Processing and Dietary Wholesomeness

### Context: processing categories

Processing improves nourishment potential and dietary flexibility, although intensive industrial processing leads to concerns (Astrup and Monteiro, 2022). This problem is captured by studies using NOVA categories: 1) unprocessed or minimally processed foods, 2) processed culinary ingredients, 3) processed foods (based on traditional methods, e.g., fermentation and pickling), and 4) ultra-processed foods (Scrinis and Monteiro, 2022).

Importantly, the difference between processed and ultra-processed foods is not just one of degree but rather one of fundamental distinction. Whereas processed foods are seen as benign when consumed in reasonable amounts, intake of ultra-processed foods is to be minimized. In addition to being highly processed, they consist of disrupted food matrices, are mostly produced from cheap, extracted, refined, and fractionated ingredients (e.g., sugars, starches, seed oils, and protein isolates), have a high degree of “artificialization” and rely excessively on additives (e.g., colorants, flavors, sweeteners, and emulsifiers), are hyperpalatable and quasi-addictive, and are typically produced by transnational corporations to create branded and profitable products, which are meant to displace other food groups (Scrinis and Monteiro, 2022).

These developments do, however, fit into broader historical trends. As opposed to ancestral-type and other traditional populations, who spent much of their day in pursuit of securing sustenance, modern (post)industrial consumers typically neither produce nor process, and often not even prepare, the foods they consume. Industrialization, upscaling of production, and increased division of labor did not only transform Western food economies over the past century-and-a-half, but also the relationship between humans and their food. Modern lifestyles created a demand for more processed ingredients, convenience foods, and ultimately ready-made meals (Table 2). Consequently, these developments have led to the replacement of the rich experiences of shared traditional meals with a plethora of individualized ready-made products, often resulting in mindless eating and the erosion of commensality.

A limitation of the NOVA classification, however, is that nutrient content is not given much attention. Minimally processed foods are generally seen as optimal, but there are major differences in nutrient variety and density between, for instance, beef, rice, kale, and watermelon. Such differences underline that the quest for adequate nourishment is contingent on the interplay between nutrient density (“Nutrient density level” section) and processing level (“Aspects of processing and dietary wholesomeness” section).

### Low levels of food processing

According to research based on the NOVA framework, minimally processed foods (category 1) are generally to be recommended (Scrinis and Monteiro, 2022). In some cases, however, unprocessed or underprocessed foods come with concerns, so that a minimum of intervention is needed. In animal-source foods, minor processing steps are often sufficient to minimize the risk of foodborne pathogens. Most concerns can be relatively easily dealt with through adequate cooking, but a warning against raw food diets needs to be issued. To deal with food safety issues in meat-derived products, rather than fresh meat, processing may be more thorough (e.g., based on salting and the use of nitrite and nitrate salts). With respect to plants, processing interventions need to be substantial in some cases, given that several plants are poorly digestible in their natural state (e.g., maize grain) or contain antinutritional factors and toxins (as for certain types of cassava and pulses). Examples include the use of boiling to remove cyanide from cassava or lima beans and lectins from beans, the destruction of trypsin inhibitor in soy upon heating, the removal of arsenic from rice by soaking, and the nixtamalization of maize to increase nutrient availability. For diets that are almost entirely plant-based, the use of nutrient fortification and individually planned supplementation schemes becomes important. The option of texturized protein is also common in plant-based strategies, especially when incorporating “alternatives” for animal-source foods. Based on these considerations, “raw vegan” diets are not advisable.

### Medium levels of food processing

Processing does not necessarily equal a lower degree of healthiness, as traditional and certain types of industrial processing techniques help to preserve or improve the innate quality of foods. Such processed foods, roughly corresponding to what is presented in the NOVA framework as category 3, have been part of traditional diets for many generations, often as geographic specialities with strong cultural connotations (e.g., traditional cheeses, fermented vegetables, and breads). In all diets, whether they are dominated by plant or animal-source foods, processing can be helpful to create distinct sensory properties and gastronomic value, preserve food quality out of season and improve shelf-life, minimize food safety risks, and improve nutrient retention and bioavailability. To achieve adequate nourishment, it is thus recommended to compose meals that are not only mostly based on whole foods (NOVA 1) and cooked using processed culinary ingredients (NOVA 2), but also complemented with processed foods (NOVA 3). This allows for the preparation of virtually all types of whole foods-based diets, while releasing a broad culinary potential and respecting culinary traditions. Such meals will be moderately processed on average, but with an improved nutrient content for some food materials and the quality of foods mostly maintained.

As argued above, processing is particularly required in diets that are heavily dominated by plants to 1) reduce the levels of plant toxins, 2) increase digestibility, 3) reduce the levels of antinutritional factors, and 4) increase the levels of specific nutrients through fortification. The latter is valuable when nutrient-dense whole foods are poorly accessible or affordable, or when such foods are avoided for reasons that go beyond nutrition. For instance, fortification may at least partially mitigate the risk of decreased musculoskeletal health when eliminating or drastically replacing animal-source foods with plant-based alternatives in the absence of careful planning and expert guidance (Webster et al., 2023). In the case of whole-food plant-based diets, diets remain within the NOVA 1 to 3 categories (when properly supplemented), but when shifting to plant-derived imitations of animal-source foods, this would likely lead to NOVA 4-dominated diets, as will be discussed below. The production of such foods typically favors ultra-processing due to the need for cosmetic additives, texturizers, and nonculinary ingredients, while also affecting the nutritional composition (Metz et al., 2023).

### Ultra-processed foods

The NOVA framework is sometimes misread as being hostile toward food processing. On the contrary, research using NOVA has restored the reputation of processed foods, by separating the types of processing that are generally beneficial from the ones that are generally harmful. The latter gives rise to ultra-processed foods (NOVA 4). Even if there may be considerable variety within this group with respect to health implications, and although the occasional consumption of certain individual ultra-processed foods may not necessarily be detrimental, diets that are dominated by ultra-processed foods are likely problematic.

The analysis of the global PURE cohort has shown a higher risk of all-cause, cardiovascular, and noncardiovascular mortality related to the consumption of ultra-processed foods (Dehghan et al., 2023). In an umbrella review, Lane et al. (2024) argued that there is “convincing evidence” to support associations between greater exposure to ultra-processed foods and risks of poorer health, spanning cardiometabolic, overweight/obesity, common mental disorder, and mortality outcomes. However, based on a GRADE certainty of evidence assessments, the majority of evidence was low to very low certainty for most of the outcomes (41 of 45 outcomes), due to the observational nature of the majority of the studies.

There are, nonetheless, more compelling reasons to restrict ultra-processed diets than the abovementioned observational evidence. In a randomized controlled trial, ultra-processed diets were shown to cause an increased caloric intake compared to unprocessed ad libitum diets, despite matching meals for calories, macronutrients, sugar, fat, sodium, and fiber (Hall et al., 2019). Diets dominated by ultra-processed foods thus seem to undermine health outside the current high fat/salt/sugar (HFSS) paradigm. The responsible mechanisms are not fully clear, but several plausible hypotheses have been put forward. Besides the fact that these foods are indeed often simultaneously high in fat and refined sugars and starch, increasing reward signals (DiFeliceantonio et al., 2018), other mechanisms seem to be at play (e.g., dilution of dietary protein driving energy overconsumption, food matrix degradation, glycaemic and inflammatory responses, and effects on gut microbiota; Scrinis and Monteiro, 2022). Moreover, ultra-processed foods are energy-dense but do not usually provide the nutrient richness and biochemical complexity of less processed foods, even when fortified with micronutrients. This may deregulate normal physiological functions, including the efficacy of dietary self-selection and natural satiety (Provenza, 2018).

Taken together, and despite the fact that observational evidence on ultra-processed foods is generally of low certainty, a conditional recommendation to avoid diets dominated by such foods may be warranted; particularly so if future health-related value and preference studies would indicate that consumers are largely willing to reduce their ultra-processed foods intake based on estimates of absolute changes in health indicators and outcomes (Zeng et al., 2023), including changes in caloric intake from the available intervention trials (Hall et al., 2019). Complete absence of ultra-processed foods is probably neither necessary nor culturally achievable, but their intake should, however, be minimized.

## Specific Target Groups with Elevated Nutritional Needs

A final consideration is that the framework presented in this study, established on general principles, may need to be developed somewhat differently for specific population groups with different needs and vulnerabilities. Nutrient requirements vary across the life course driven by different biological processes such as growth or reproduction. Dietary guidelines may distinguish these requirements as special considerations in dietary patterns, calling out specific foods or nutrients that support or undermine these processes (Herforth et al., 2019; U.S. Dietary Guidelines 2020). The consequences of not meeting these nutritional needs, for example during the first 1,000 d of life, can be dire and life-long, leading to stunted growth, weakened immune systems, and impaired cognitive development. Certain life stages have increased protein and priority micronutrient density requirements: infants and young children, pregnant and lactating women, women of reproductive age, and older adults. The new WHO Guideline for complementary feeding specifically recommends daily consumption of animal-source foods for children 6 to 23 mo, as these nutrient-dense foods help to bridge the nutritional gaps that might not be adequately covered by breast milk and plants alone after the first 6 mo of life (WHO, 2023). The UN Food and Agriculture Organization recently published an extensive report synthesizing the evidence base for the contribution of animal-source foods to human health, highlighting the need for these foods in particular life stages (FAO, 2023). In low-income countries, the dietary framework proposed in the present article may also need to put more emphasis on affordability, possibly requiring a more prominent role for effective food processing interventions, including fortification. Providing people with livestock and appropriate training to make use of available resources is another alternative to enhance food security and diet adequacy. Such considerations would require their own dedicated analysis, which is beyond the scope of the present study.

## Conclusion

Dietary recommendations give primacy to the increasingly eroded concept of “healthy diets,” often dictated by consensus-based findings from the discipline of nutritional epidemiology, and its interpretation of the evidence for preventing noncommunicable diseases. Such evidence may overshadow the need for adequate nourishment and health-related values and preferences of the target populations in a broader sense. Future dietary guidelines may benefit from a more flexible process that embraces evidence-based practice and provides more space and flexibility for dietary habits and patterns within the omnivorous spectrum. Flexibility nonetheless comes with limits, outside of which dietary solutions risk becoming nutritionally inadequate and harmful to health. Both nutrient density, mirrored to a substantial degree in the animal–plant ratio, and the degree of food processing pertain to this discussion. While consciously refraining from formulating more specific guidance than what is provided by cultural and physiological “nutritional wisdom,” we contend that self-selection of dominantly nutrient-dense and satiating foods, which are ideally of a mostly minimally and moderately processed nature (corresponding to the green zone in Figures 1 and 2), maximizes dietary flexibility according to personal needs and preferences within a broad yet optimal domain for adequate nourishment. How such dietary advice and guidelines would compare to conventional recommendations in meeting nutrient security and avoidance of chronic diseases needs to be further explored, especially for specific population groups.

**Table 2.** Ultra-processed foods as percent of total calories

Percent	Data per region
70+	Highest quintile of consumers in the United Kingdom, Umited States, and Australia (70% to 80%)
40 to 69	Most high-income countries on average Children in the United Kingdom and United States (55% to 67%) and France (46%)
15 to 39	French adults (35%) Low- and middle-income countries on average (18% to 35%)
0 to 14	Individual consumers adopting whole-food diets Ancestral-type diets

After Khandpur et al. (2020), Fardet et al. (2021), and Scrinis and Monteiro (2022).

## About the Authors

 **Leroy Frédéric** graduated as a bioengineer (Ghent University, 1998) and obtained a PhD in applied biological sciences at the Vrije Universiteit Brussel (VUB, 2002), where he now holds a professorship in food science and (bio)technology. He is primarily affiliated with the research group of Industrial Microbiology and Food Biotechnology (IMDO) and, additionally, with the research group of Interdisciplinary Historical Food Studies (FOST). His research deals with animal-source foods, food processing and technology, food fermentation and microbiology, human nutrition, food systems and sustainability, and interdisciplinary “food studies.” **Corresponding author**: Frederic.Leroy@vub.be

 **Ty Beal** is a global nutrition and food system scientist dedicated to achieving healthy and sustainable diets for all. Through his research, he examines dietary patterns and their impacts on human health and the environment. As a research advisor at the Global Alliance for Improved Nutrition (GAIN), he helps shape evidence-based programs and policies. Dr. Beal received his PhD in geography with an emphasis in global nutrition from the University of California, Davis.

 **Nel de Mûelenaere** is assistant professor in contemporary history at the Vrije Universiteit Brussel, where she is affiliated with the Interdisciplinary Historical Food Studies (FOST) research center. She held visiting fellowships at UC Berkeley (2022) and Cornell University (2019) and was the academic co-director of the Brussels Institute for Advanced Studies (BrIAS; 2020 to 2022). For BrIAS, she convened a program on food systems and green deal policies in and beyond Europe (2022) and on the future and past of food crises (2023). She has published on food policies and international development in the Interwar Period.

 **Stefaan De Smet** is a full professor in Animal Science at the Faculty of Bioscience Engineering, Ghent University, Belgium. He graduated as a bioengineer (Ghent University, 1987) and obtained a PhD in agricultural sciences (Ghent University, 1993). His research deals with the intrinsic quality traits of animal-source foods, particularly meat, related to animal nutrition and production factors and with a strong interest in the impact on human nutrition and health.

 **Bradley Johnston** is a PhD methodologist and an associate professor in the Departments of Nutrition (primary appointment), and Epidemiology and Biostatistics at Texas A&M University. His work involves health status measurement, and the application of randomized trial, systematic review, meta-analysis, and clinical and public health guideline recommendation methods to a wide range of areas, with a particular interest in evidence-based nutrition practice. As the director of EvidenceBasedNutrition.org, he leads an international consortium of researchers developing high-quality, value-added systematic reviews and guideline recommendations on clinical and public health nutrition questions.

 **Frits Heinrich** was trained as an ethnoarchaeobotanist and agricultural and food historian at the University of Groningen and University of Oxford, and obtained a PhD in history at the Vrije Universiteit Brussel. He holds an interdisciplinary assistant professorship in Ancient, Agricultural, and Food History at the research groups Interdisciplinary Historical Food Studies (FOST) and Industrial Microbiology and Food Biotechnology (IMDO) at the Vrije Universiteit Brussel. Frits’ interdisciplinary research is situated at the intersection of ethnoarchaeobotany, history, food chemistry, and biotechnology. His historical research focuses on Roman agriculture, diet, and nutrition. Frits is also interested in traditional agricultural and food practices and conducts historical farming experiments as well as ethnobotanical and archaeobotanical fieldwork projects.

 **Lora Iannotti** is a professor of public health and founding director of the E3 Nutrition Lab at Washington University in St. Louis. Her lab aims to identify nutrition solutions that embrace principles embodied in the three E’s: equity, environment, and evolution. Dr. Iannotti leads projects in Ecuador, Haiti, and Madagascar where she collaborates with local partners to test innovative approaches to achieving sustainable, healthy dietary patterns that improve young child growth and brain development. Her research related to animal-source foods has informed the global discourse on nutrition equity, climate change, and planetary boundaries. Dr. Iannotti is the Director of Planetary Health for the newly launched Center for the Environment at Washington University in St. Louis. Iannotti received her doctorate from the Johns Hopkins University Bloomberg School of Public Health, and a Master of Arts degree in Foreign Affairs from the University of Virginia.

 **Neil Mann** retired after 7 yr as professor and Head of Food Science and Human Nutrition in the School of Agriculture and Food Science, at the University of Melbourne. He is now honorary professor of Human Nutrition at the School of Exercise and Nutrition Science of Deakin University Australia. His qualifications include BAppSc in chemistry from the Victorian Institute of Colleges and a BSc(Hons) in biochemistry, from Deakin University Australia. He has a PhD in nutritional biochemistry from Deakin University, and is a fellow of the Nutrition Society of Australia. He previously held the position of professor of Nutritional Biochemistry and head of Food and Nutrition at RMIT University Melbourne for 12 years, prior to which he spent 10 yr as associate professor at RMIT. He has over 35 yr of experience in nutrition, food, and agriculture teaching and research, coordinating clinical trials in the areas of long-chain fatty acids, obesity, diabetes, hunter-gatherer diets, and aspects of metabolic syndrome.

 **Andrew Mente** received his doctoral degree in epidemiology from the University of Toronto. He is currently an associate professor at the Population Health Research Institute (PHRI) at McMaster University in Hamilton, Ontario, Canada. Dr. Mente has studied dietary nutrients, foods, and patterns and their role in cardiovascular diseases. He has co-led an investigation on the role of fats and carbohydrates in cardiovascular disease in 135,000 participants in 21 countries in 5 continents in the PURE cohort. This work has challenged long-standing wisdom on diet and health. Recently, Dr. Mente and his team developed a healthy diet score that is associated with health outcomes and is globally applicable using data from PURE and replicated it in 5 independent studies on a total of 245,000 people from 80 countries. This is the largest and most diverse study ever on diet and health outcomes.

 **Alice Stanton** is a clinician-scientist. Her current position is professor of cardiovascular therapeutics at the RCSI University of Medicine and Health Sciences, qualifications include MB (medicine, University College Dublin), BSc (physiology, University College, Dublin), Post-grad Diploma (statistics, Trinity College Dublin), PhD (pharmacology, University College Dublin), and FRCPI (medicine, Royal College of Physicians of Ireland). She is currently most interested in evidence-based healthy diets from sustainable food systems. Other research interests include nutrition, climate change, lifestyle medicine, cardiovascular therapeutics, genetics, pharmacogenetics, clinical imaging, ambulatory blood pressure monitoring, and hypertension.
